# Novel anticancer agent, SQAP, binds to focal adhesion kinase and modulates its activity

**DOI:** 10.1038/srep15136

**Published:** 2015-10-12

**Authors:** Jesus Izaguirre-Carbonell, Hirofumi Kawakubo, Hiroshi Murata, Atsushi Tanabe, Toshifumi Takeuchi, Tomoe Kusayanagi, Senko Tsukuda, Takeshi Hirakawa, Kazuki Iwabata, Yoshihiro Kanai, Keisuke Ohta, Masahiko Miura, Kengo Sakaguchi, Sachihiro Matsunaga, Hiroeki Sahara, Shinji Kamisuki, Fumio Sugawara

**Affiliations:** 1 Department of Applied Biological Science, Faculty of Science and Technology, Tokyo University of Science, 2641 Yamazaki, Noda, Chiba 278-8510, Japan; 2 Laboratory of Biology, Azabu University School of Veterinary Medicine, Sagamihara 229-8501, Japan; 3 Department of Oral Radiation Oncology, Graduate school, Tokyo Medical and Dental University, Bunkyo-ku, Tokyo 113-85-10, Japan

## Abstract

SQAP is a novel and promising anticancer agent that was obtained by structural modifications from a natural compound. SQAP inhibits angiogenesis *in vivo* resulting in increased hypoxia and reduced tumor volume. In this study, the mechanism by which SQAP modifies the tumor microenvironment was revealed through the application of a T7 phage display screening. This approach identified five SQAP-binding proteins including sterol carrier protein 2, multifunctional enzyme type 2, proteasomal ubiquitin receptor, UV excision repair protein and focal adhesion kinase (FAK). All the interactions were confirmed by surface plasmon resonance analysis. Since FAK plays an important role in cell turnover and angiogenesis, the influence of SQAP on FAK was the principal goal of this study. SQAP decreased FAK phosphorylation and cell migration in human umbilical vein endothelial cells and A549 cancer cells. These findings suggest that inhibition of FAK phosphorylation works as the mechanism for the anti-angiogenesis activity of SQAP.

Angiogenesis is a physiological process that promotes the formation of new vascular segments originating from existing vessels, such as capillaries and venules. It is a normal and vital process during growth and development; however, it is also a fundamental step in the transition of tumors from a dormant state to a malignant one^1^. In the tumor environment, angiogenesis often results in relatively incomplete capillaries which allow tumor growth at the periphery and hypoxia at the center, leading to necrosis^2^. Folkman *et al.* proposed that tumor growth and metastasis were angiogenesis-dependents and hence suggested blocking angiogenesis as a strategy to arrest tumor growth^3^. Subsequently, it was proposed that cells in precancerous tissue acquired angiogenic capacity while becoming cancerous^4^. Angiogenesis has become a well-accepted target for cancer prevention and thus, many angiogenesis inhibitors have been developed^5^. Our group has focused on the identification of new antiangiogenic agents through collection and screening of natural products.

Sulfoquinovosyl diacylglyceride (SQDG) is a common secondary metabolite that is found in photosynthetic bacteria, algae, and higher plants^6^. A sulfoquinovosyl monoacylglyceride (SQMG) analogue was isolated from the small intestine of sea urchins as result of natural products research^7^. We reported that SQMG significantly inhibited tumor growth of lung and colon adenocarcinomas transplanted in nude mice in combination with X-irradiation^7^,^8^. Mori *et al.* found that the SQMG antitumor effect was involved in antiangiogenesis by mediating *Tie2* gene downregulation^9^. Additionally, the agent was reported to upregulate thrombospondin 1 (TSP-1) *in vivo* and *in vitro*^10^. In our laboratory, SQMG was chemically modified to sulfoquynovosylacylpropanediol (SQAP) (Fig. 1) via organic synthesis^11^.

SQAP is a promising radiosensitizing agent for anticancer therapy. The ability of SQAP to trigger antiangiogenesis *in vivo* with very few side effects, similar to SQMG, has been confirmed in previous biological studies. Moreover, we observed that combination therapy with indirect ionization against human cancers transplanted in mice synergistically enhanced tumor arrest via an antiangiogenic effect^12^ (see Supplementary Fig. S1). However, the radiobiological mechanisms that modify the tumor microenvironment are still unknown. In this paper, we aimed to reveal these mechanisms.

In order to elucidate the mechanism of action of a compound, the identification of target proteins has become a standard approach in biology^13^. T7 phage display is an effective technique to identify proteins that bind to small molecules of interest in cell-free experiments. This method was originally developed in 1985 by GP Smith^14^. Sche *et al.* incorporated cDNA into phage particles, allowing to express transcript of every gene from any desired cell type^15^. The encoded peptides are expressed or “displayed” on the phage particle surface as a fusion product with one of the phage coat proteins. Every phages particles displays peptides in a diverse range of sizes up to about 1200 amino acids (aa) on their capsids. These peptides are equivalent to proteins or to their corresponding fragments encoded in living cells or organs^16^. The method requires biotinylated small molecule derivatives immobilized on an avidin-coated microplate to screen interactions. Phage libraries are subjected to a selection step in which the small molecule binding the phage particles are isolated and gradually enriched through further rounds of selection. By sequencing the phage DNA encoding the binding displayed peptide it is possible to determine specific and selective ligands to target receptors^17^ and more importantly, the putative binding site. In recent years, our group has identified many binding partners using T7 phage display technology^18^,^19^,^20^,^21^.

In this study we report the identification of five SQAP-binding proteins using the T7 phage display method: sterol carrier protein 2 (SCP-2), multifunctional enzyme type 2 (MFE-2), proteasomal ubiquitin receptor (ADRM1), UV excision repair protein (HR23B) and focal adhesion kinase (FAK). All the interactions were validated via surface plasmon resonance (SPR). FAK is a well-known tyrosine kinase that plays a critical role in angiogenesis during embryonic development and cancer progression^22^,^23^. Moreover, FAK phosphorylation is increased after radiation exposure in lung cancer^24^. Since SQAP anticancer activity is based on antiangiogenesis and radiosensitization, the relationship between FAK and SQAP was further studied using a docking simulation and cell based assays. SQAP inhibited vascular endothelial growth factor (VEGF)-dependent FAK phosphorylation in human umbilical vein endothelial cells (HUVEC) as well as fibronectin-dependent FAK phosphorylation in A549 lung cancer cells. Downstream targets phosphorylated by FAK including paxillin and Akt were likewise inhibited. Moreover, we demonstrated that SQAP arrests cell migration, a process dependent on phosphorylated FAK and a crucial step for angiogenesis. The obtained data suggest that SQAP antiangiogenic activity may be due to the modulation of FAK phosphorylation.

## Results

### Indentification of SQAP-binding proteins using a T7-phage display screen

**Figure 1:**
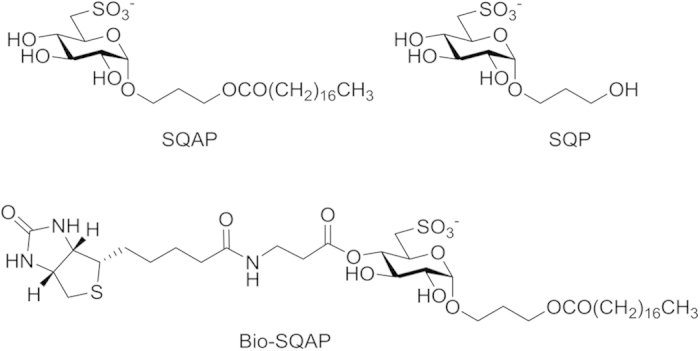
Structures of SQAP, biotinylated SQAP and SQP.

**Figure 2:**
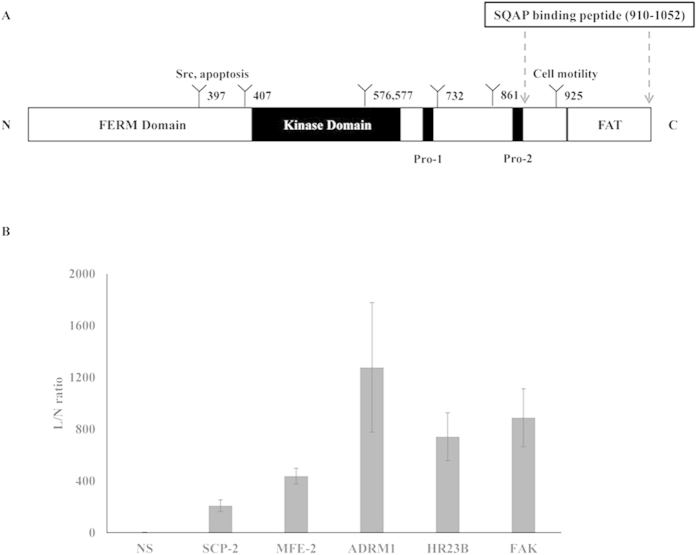
Affinity study of identified SQAP binding phage clones. (**A**) Schematic representation of full-length FAK. The region that corresponds to the 142 aa sequencedisplayed on the SQAP-binding T7 phage clone is marked. **(B)** Each single T7 phage clone sequence displayed the binding peptides or a control phage clone (NS phage) was incubated in a nonimmobilized well (control) or in a SQAP-immobilized well (SQAP). The recovery rate was calculated by dividing the phage titer obtained from the eluate by the input phage titer. Then, the recovery rate was compared between control and SQAP wells to obtain the ligand vs non ligand (L/N rate).

To identify SQAP-binding proteins, we performed a T7 phage display screen. Lung tumor, human cervical carcinoma (HeLa) and HUVEC phage libraries were selected because they are well studied models for anticancer and antiangiogenesis research. We immobilized biotinylated-SQAP (Bio-SQAP, Fig. 1) on a streptavidin-coated well followed by incubation with the corresponding phage libraries. After sufficient time for interactions, the unbound phage particles were washed out, and those binding to SQAP were recovered and amplified for future rounds of analysis. The ratio of phage particles recovered (eluted phages/input phages) greatly increased after four to six round of selections (see Supplementary Fig. S2), suggesting an enrichment of potential SQAP-binding phages particles. Sixteen samples were randomly extracted and the DNA sequences of the displayed peptides were determined. Homology search performed on FASTA demonstrated that the screened peptides could be classified into three types; SCP-2 and MFE-2 obtained from lung tumor and the HUVEC phage library, ADRM1 and HR23B obtained from the HeLa library and FAK obtained from the lung tumor library (Table 1, Fig. 2A). Interestingly, we obtained SCP-2 and MFE-2 across two different phage libraries and the binding region in both cases corresponded to a SCP-2 domain.

To confirm the affinity to SQAP of the peptides obtained from the phage display, we amplified each phage clone and a control phage displaying no peptide (NS) and applied them to the SQAP-immobilized or nonimmobilized well. The ratio of recovered phage particles from the SQAP-immobilized well was compared with that from the nonimmobilized control well. The phage particle recovery from SQAP-immobilized wells was dramatically higher than that from the control wells (Fig. 2B), and the NS phage particles did not bind to SQAP. These results highlighted the selective interaction between SQAP and the peptides.

### SPR analysis

**Figure 3:**
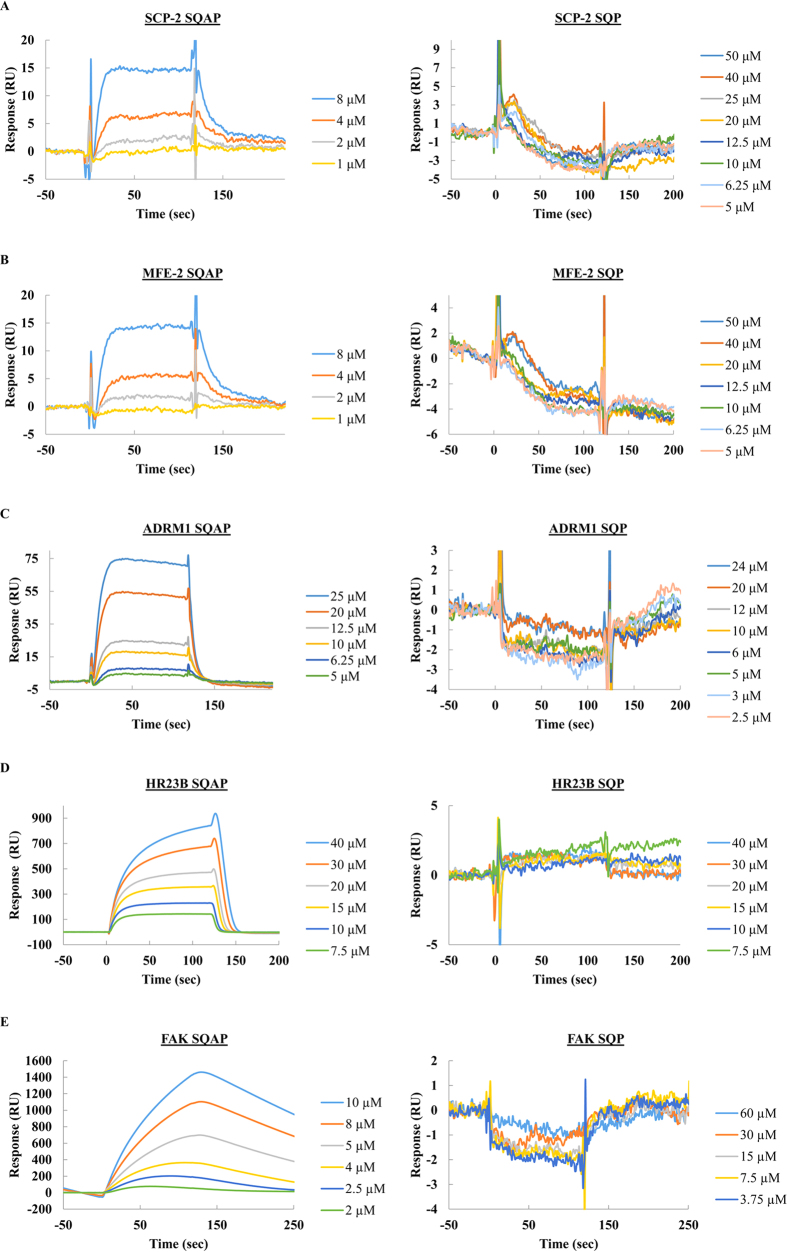
SPR analysis of the binding between SQAP and target proteins. Sensorgrams obtained by SPR analysis of SQAP (left) and SQP (right) with the candidate proteins: (**A**) SCP-2 *K*_D_ 15.6 ± 2.2 μM, (**B**) MFE-2 *K*_D_ 14.2 ± 1.79 μM, (**C**) ADRM1 *K*_D_ 9.65 ± 2.91 μM, (**D**) HR23B, (**E**) FAK *K*_D_ 3.24 ± 0.21 μM. The candidate proteins were immobilized onto the surface of a CM5 sensor chip. A solution of SQAP or SQP at variable concentrations was injected to generate result binding responses (RU) recorded as a function of time (sec). The results were analyzed using BIA evaluation 4.1.

SPR was utilized as an independent confirmation of the kinetic parameters between SQAP and the putative binding proteins. We prepared His-tagged truncated forms of SCP-2, MFE-2 and FAK corresponding to the regions displayed on the SQAP-binding phages 400-547 aa, 598–736 aa and 910–1052 aa respectively. The recombinant proteins were then expressed in *Escherichia coli* and purified to homogeneity. In addition, full length, His-tagged ADRM1 and GST-tagged HR23B (GST-HR23B) were also used. The proteins were first immobilized on a CM5 sensor chip of the SPR biosensor (Biacore 3000). For GST-HR23B analysis, a GST protein was additionally immobilized and used as a control in order to eliminate possible nonspecific binding of SQAP. SQAP samples at different concentrations were then injected over the proteins immobilized on the sensor chip. As a negative control we used sulfoquynovosylpropanediol (SQP), which lacks the fatty acid moiety of SQAP (Fig. 1). The SPR data (Fig. 3) was subsequently analyzed to determine the dissociation constants (*K*_D_) which were estimated to be 15.6 μM for SCP-2, 14.2 μM for MFE-2, 9.65 μM for ADRM1 and 3.24 μM for FAK. SQAP presented a weak nonspecific binding to GST which prevented kinetic analysis for HR23B. Our results showed a specific binding response of SQAP in a dose-dependent manner for all the analyzed proteins.

### Docking simulation between FAK and SQAP

**Figure 4:**
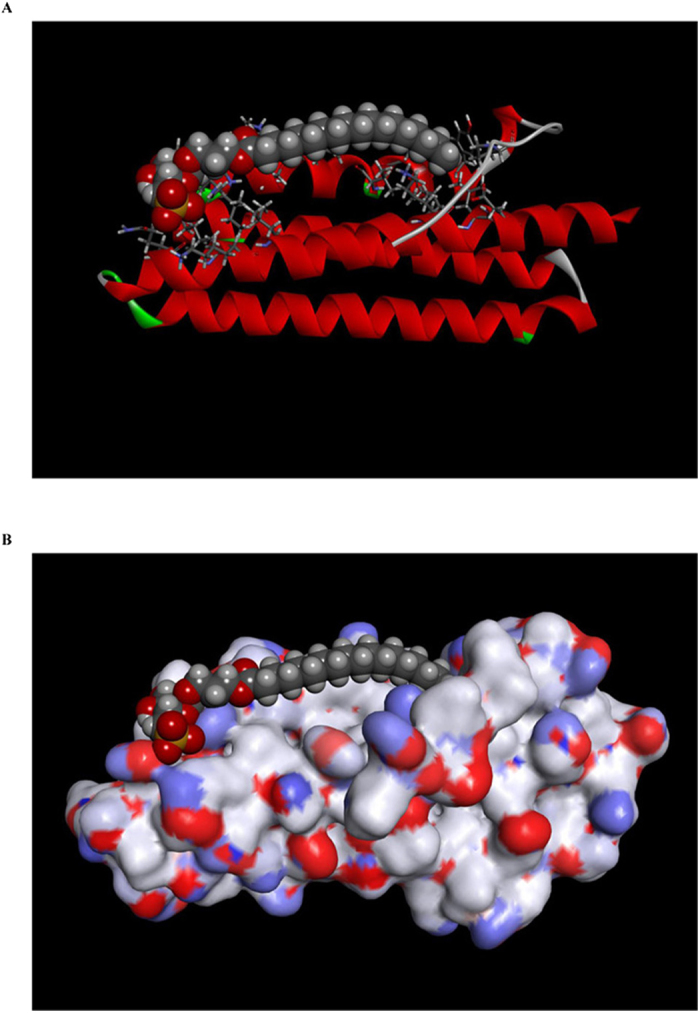
Docking simulation. Calculated binding model of SQAP-FAK complex are shown as a molecular surface (**A**) and in ribbon format (**B**).

We selected FAK for further research because of its involvement in angiogenesis in endothelial cells^25^ and its role in development of human cancer^26^. To further validate the binding of SQAP to FAK, we carried out a docking simulation analysis on SQAP employing the focal adhesion targeting (FAT) domain (906–1047) of FAK taking into account that SQAP showed affinity with a peptide fragment corresponding to FAT domain (910–1052). FAT domain from FAK has been already reported^27^. The docking simulation indicated that the sulfone group of SQAP can interact with side chains of residues, N943, N1014 and K1018 of FAK, and the fatty acid moiety of SQAP can interact with side chains of residues, Y925, V932 and K1032 of FAK (Fig. 4A,B).

### SQAP inhibits HUVEC and A549 cell migration through the inhibition of FAK phosphorylation

**Figure 5:**
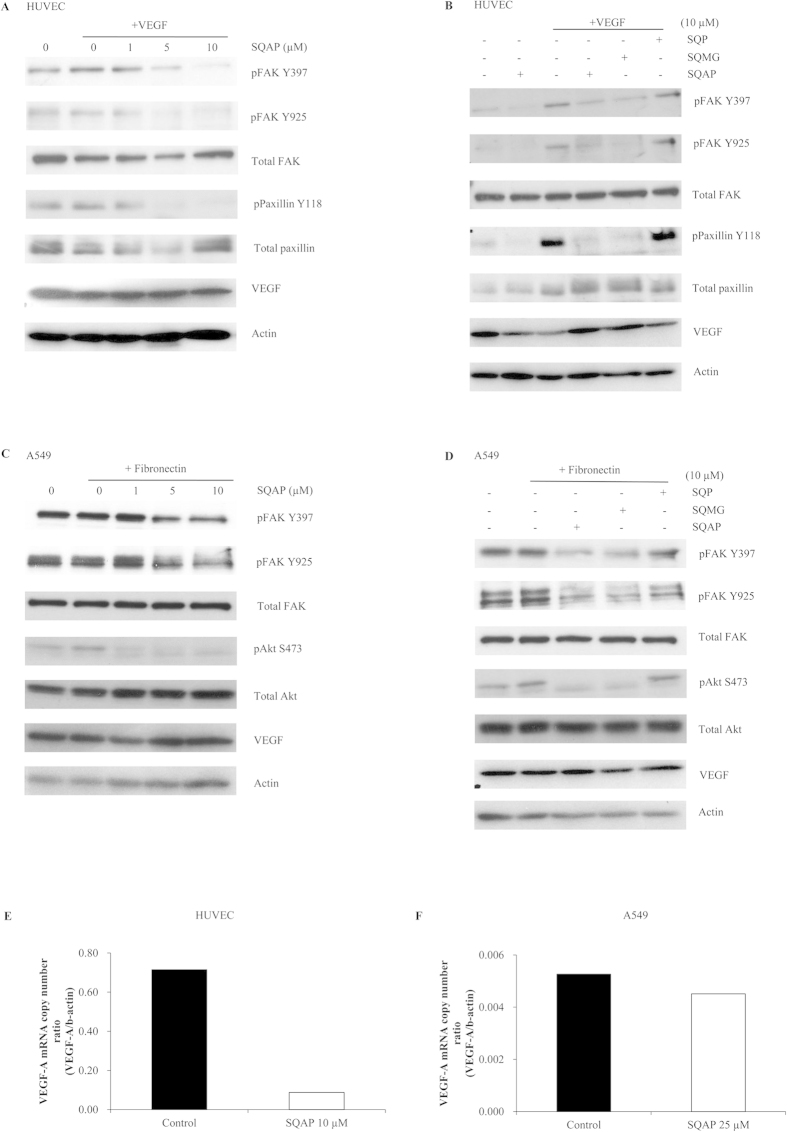
Effect of SQAP on FAK phosphorylation. The level of FAK phosphorylated at Y397 and Y925 was evaluated by Western blotting. (**A**) HUVEC cells were serum-depleted for 20 h before treatment with SQAP or vehicle for 30 min followed by treatment with or without VEGF (50 ng/mL) for 30 min. (**B**) HUVEC cells were cultured as stated in section A and then treated with SQAP, SQMG or SQP. (**C**) A549 cells were serum-deprived for 48 h before pretreatment with SQAP or vehicle followed by treatment with or without fibronectin (10 μg/mL) for 30 min. (**D**) A549 cells were cultured as stated in section C and then treated with SQAP, SQMG or SQP. (**E–F**) HUVEC and A549 cells were treated with SQAP for 6 h before adding an RNA stabilization reagent and prepared for Quantitative real-time RT-PCR.

Formerly, it was reported that FAK is phosphorylated and hence activated in response to VEGF in endothelial cells^28^. The phosphorylation of FAK in response to VEGF plays an important role in the regulation of endothelial cell migration during angiogenesis. Therefore we hypothesized that SQAP activity could potentially be mediated through binding to FAK. Our phage display data showed that SQAP binds to FAK at the 910–1052 aa region (Table 1, Fig. 2A). In this region, FAK interacts with paxillin through FAT domain and becomes autophosphorylated on Y397^29^,^30^. Y397 is an autophosphorylation site which is crucial for FAK activity and has been related to apoptosis and tumor growth^31^,^32^. FAK is also phosphorylated at Y925, leading to the regulation of adhesion turnover and cell motility^33^. Thus we chose to study the FAK phosphorylation on Y397 and Y925 residues. Initially, we examined whether SQAP affected phosphorylation of FAK in HUVEC cells stimulated with VEGF in the presence or absence of SQAP. As shown in Fig. 5A, the phosphorylation level of FAK at Y397 and at Y925 after VEGF stimulation was appreciably reduced by SQAP after 1 h in a dose-dependent manner. Since SQMG was previously reported to bind to VEGF^34^, we could not rule out the possibility of a binding between SQAP and VEGF so we analyzed this interaction by SPR. We observed that SQAP did bind VEGF (data not shown) suggesting that the SQAP-mediated reduction of FAK phosphorylation might originate from its interaction with VEGF. Therefore, we investigated whether SQAP could inhibit FAK phosphorylation independently of VEGF. We analyzed FAK phosphorylation in the absence of VEGF and observed that SQAP inhibited the phosphorylation of FAK signal within 1 h in a VEGF independent manner (Fig. 5B). Additionally, we treated HUVEC with VEGF in the presence of SQMG and SQP. The obtained data shows that both SQAP and SQMG can inhibit FAK phosphorylation, but not SQP, which is consistent with the SPR data.

Furthermore, we looked at FAK signaling pathway to validate our results. Paxillin binds to FAK 919–938 residues which is a sequence contained within the region where SQAP is predicted to bind. Binding between paxillin and FAK results in paxillin phosphorylation at Y118^35^,^36^. Thus, we examined paxillin phosphorylation. The reduction in FAK phosphorylation achieved by treatment with SQAP in HUVEC correlated with the inhibition of paxillin-phosphorylation without altering total paxillin levels (Fig 5A,B). Additionally, we studied Akt phosphorylation at S473 on account of FAK is a known factor in control of Akt activity^37^. We observed that Akt phosphorylation was diminished when treated with SQAP or SQMG for 21 h (see Supplementary Fig. S3).

Fibronectin is a glycoprotein located in the extracellular matrix that binds to integrins. Previously it was reported that A549 lung epithelial cells stimulated by fibronectin showed incremental FAK phosphorylation^38^. A549 lung cancer cells have been used before for studying cell migration because of their metastatic model potential^39^. A549 cells are also one of the tumor cell lines sensitive to SQMG^9^. Therefore, we studied the phosphorylation of FAK in A549 cells (Fig. 5C,D). SQAP could inhibit the phosphorylation of FAK at Y397 and Y925 after 1 h treatment in a dose dependent manner. Furthermore, SQP failed to inhibit FAK phosphorylation on A549 cells (Fig. 5D). We also examined paxillin and Akt phosphorylation in A549 as downstream targets of FAK. While Akt phosphorylation at S473 was clearly downregulated after SQAP treatment (Fig. 5C,D) inhibition of paxillin phosphorylation at Y118 took 21 h treatment (see Supplementary Fig. S3). We hypothesize that SQAP diminish on Akt phosphorylation being more readily in A549 cancer cells than in HUVEC endothelial cells might be due to a higher Akt basal activity on cancer cells.

Interestingly, it was previously reported that a point-mutation in FAK Y925 phosphorylation disrupted FAK ability to promote VEGF expression^40^. Consequently we checked VEGF mRNA expression using quantitative real-time RT-PCR. While VEGF production level was reduced after 6 h SQAP treatment on HUVEC cells, A549 required an increased concentration of SQAP that lead to a mild production decrease (Fig. 5E,F). Western blot data did not show remarkable differences after 1 h treatment (Fig. 5A–D). We concluded that SQAP mediated FAK Y925 phosphorylation decrease might arrest VEGF production. Altogether we could observe that SQAP inhibits FAK phosphorylation in HUVEC and A549 cells which correspond to antiangiogenesis and anticancer activity resulting in disruption of downstream signaling.

**Figure 6:**
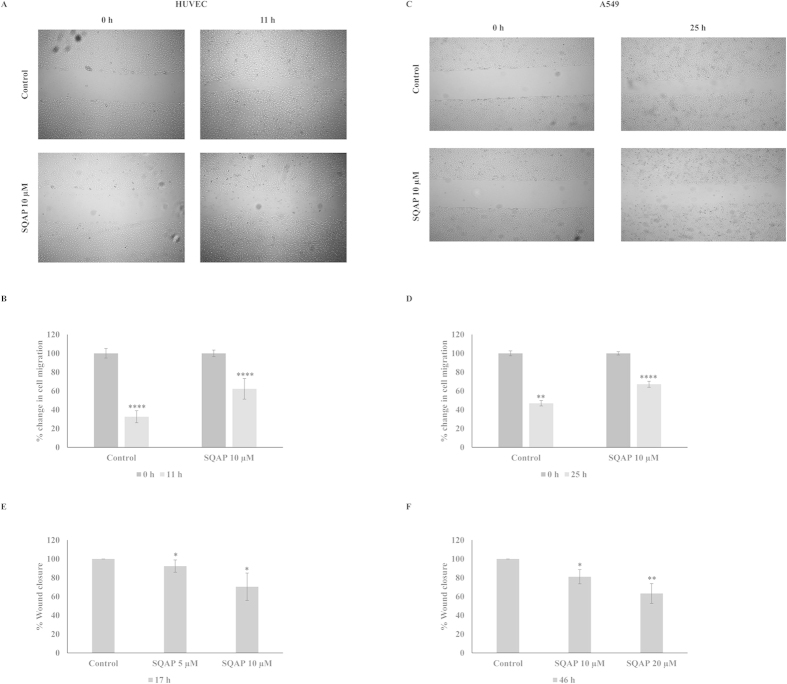
SQAP inhibits cell migration *in vitro*. (**A**) HUVEC cells were seeded and grown to 100% confluence in 60-mm diameter dishes. After generating a single scratch in the monolayer, the cells were cultured for 11 h in medium containing 1% FBS and VEGF (50 ng/mL) in the presence or absence of SQAP. Photographs were taken immediately after 11 h. (**B**) The change in wound width was measured as described in the Materials and Methods. (**C**) A549 cells were seeded and grown to 100% confluence in 60-mm diameter dishes. After generating a single scratch in the monolayer, the cells cultured for the 25 h in media containing 1% FBS and fibronectin (10 μg/ml) treated with SQAP or vehicle. Photographs were collected immediately after wounding and after 25 h. (**D**) The change in wound width was measured as described in the Materials and Methods. Cell migration was additionally examined in 96 well plates. HUVEC (**E**) and A549 cells (**F**) were seeded and grown into confluence overnight. After removing the stoppers, the live cells were stained with CellTracker^TM^ Green and incubated for the indicated time. The fluorescent emission signal was obtained with a plate reader. Values given as mean ± std; significance is indicated relative to control parent population, *P < 0.05, **P < 0.01, ***P < 0.001, ****P < 0.0001.

It is possible that depending on the site of FAK tyrosine phosphorylation distinct signaling complexes may occur, thus producing independent biological effects. For example, it was previously reported that the overexpression of Y397F-mutated FAK mainly induced apoptosis, while both Y397 and Y925 phosphorylation are related to cell turnover^33^,^41^. Given that SQAP could inhibit Y397 and Y925 phosphorylation, we sought to characterize the role of SQAP on HUVEC and A549 cells. Using an *in vitro* wound healing assay, we observed that SQAP was able to decrease HUVEC (32 vs 62% wound size) and A549 (46 vs 68% wound size) cell migration rate into the wounded area (Fig. 6A–D). Moreover, we tried to reproduce these results by using an *in vitro* cell migration assay (Fig. 6E,F). Indeed HUVEC and A549 cell migrations were arrested by SQAP. To rule out the possibility that inhibition of the cell migration by SQAP was due to cytotoxicity, a WST-8 assay was performed. SQAP exhibited noncytotoxic effects against either HUVEC or A549 cells (see Supplementary Fig. S4). Furthermore, cell survival of SQMG on tumor and endothelial cells was studied previously^42^. Together, these results suggested that SQAP arrests migration affecting FAK phosphorylation.

## Discussion

Our laboratory has been interested for a long time in phage display assays, which provides great potential for uncovering binding targets of small therapeutic molecules^43^. By using this method we were able to screen rapidly SQAP against phage libraries containing up to 10^10^ variants instead of utilizing a time and labor intensive approach of genetically modifying and analyzing individual proteins or peptides. Multiple rounds of selection enabled phage particles enrichment and allowed us to obtain five candidate proteins regardless of their solubility or protein cell expression levels. Phage display led to the identification of SCP-2 and MFE-2 in two independent screenings (lung tumor and HUVEC phage libraries). More interestingly, the SQAP-binding regions of both proteins correspond to an SCP-2 domain, facilitated with phage display as a method that provides high reliability within a great scope of binding targets. The affinity of each SQAP-binding proteins could be reproduced in two independent experiments including affinity studies for individual T7 phages and SPR.

SQAP contains an 18 carbon long fatty acid chain that resembles most important biological fatty acids like stearic, oleic and linoleic acids. We hypothesized that this fatty acid chain could confer SQAP numerous biological binding abilities. Indeed, by T7 phage display we found a heterogeneous group of SQAP-binding proteins which were related to lipid metabolism, DNA repair complexes, ubiquitin pathway or cell turnover. Furthermore, we compared those bindings using SPR and analyzed FAK phosphorylation associated with SQAP or SQP. Since SQP neither bound to SQAP-binding proteins nor affected FAK phosphorylation, we concluded that the presence of the SQAP fatty acid chain is essential for its activity and that SQAP might not address only one but several targets.

One of these targets, FAK, was the most promising among the SQAP-binding proteins obtained from the T7 phage display because of its crucial role in angiogenesis. Previously it was reported that TSP-1 induces phosphorylation of FAK at Y397 in vascular smooth muscle cells and in endothelial cells^44^,^45^. Our group reported that SQMG treatment on diverse cell lines including A549 and HUVEC resulted in the upregulation of the *TSP-1* gene^10^. Interestingly, phosphorylation of Y397 and Y925 of FAK have been proposed to mediate TSP-1 induced PI3-kinase activation^45^. Taking into account that TSP-1 expression levels were upregulated after using the PI3-kinase inhibitor LY 294002 in endothelial cells^46^ we theorize that the inhibition of FAK phosphorylation by SQAP and SQMG might arrest PI3-kinase activation which in turn will upregulate TSP-1. This relationship is currently under investigation.

Recently, Farnie G. *et al.* suggested that cancer stem cells (CSCs)-enriched populations showed increased FAK activity and accordingly radioresistance^47^. They successfully used the FAK inhibitor PF573228 in combination with radiotherapy to block the formation of clumps of breast CSCs. SQAP activity resembles PF573228 resulting in radiosensitizing activity and FAK as a molecular target. Hence, SQAP binding to FAK may not only result in reduction of tumor invasion but also improve sensitivity to radiotherapy.

We have demonstrated here that SQAP is a novel potential pharmaceutical inhibitor of FAK phosphorylation on Y397 and Y925 in HUVEC and A549 cells. Autophosphorylation at Y397 is essential for the binding of tyrosine kinase Src and for Y925 phosphorylation^48^. Besides, the activity of FAK is linked to Y397 and Y925 phosphorylation, which control cell migration^33^. The reduction of Y397 and Y925 phosphorylation on FAK had an impact on Akt and paxillin phosphorylation and likely reduced VEGF production. All in all this data was sustained by the cell migration data where SQAP could inhibit cell migration in a wound healing assay.

Taken together, our results revealed that SQAP directly bound to SCP-2, MFE-2, ADRM1, HR23B and FAK. Moreover, SQAP reduced the phosphorylation levels of FAK at Y397 and Y925. Thus, the interaction between SQAP with FAK and subsequent reduction of FAK phosphorylation may explain the antiangiogenic and radiotherapy synergistic activities of SQAP. We believe that SQAP is a valuable tool for investigating the molecular functions of FAK and is a promising agent for the treatment of tumor-induced angiogenesis.

## Materials and Methods

### Synthesis of biotinylated-SQAP

Biotinylated-SQAP was synthesized in our laboratory using the same procedure described before for biotinylated-SQMG^49^. ESIMS: *m/z* 864 [M – H]^–^.

### Antibodies and reagents

Mouse monoclonal anti-actin (clone C4), rabbit polyclonal anti-p-FAK (Y397) and mouse monoclonal anti-FAK (Clone 4.47) were purchased from Millipore (Billerica, MA, USA), rabbit polyclonal anti-SCP-2 was from Abcam (Cambridge, UK) and rabbit polyclonal anti-p-FAK (Y925) was obtained from Santa Cruz Biotechnology (Santa Cruz, CA, USA). Rabbit polyclonal anti-p-Paxillin (Y118), anti-Paxillin, anti-Akt (S473) and anti-Akt were purchased from Cell signaling (MA, USA). Human full length His-tagged ADRM1 (His-ADRM1) and GST-tagged HR23B (GST-HR23B) proteins were purchased from ATgen (Gyeonggi-do, Korea) and Abnova (Taipei, Taiwan) respectively.

### Cell culture conditions

A549 cells were obtained from Health Science Research Resources Bank (Sendai, Japan) and maintained in Dulbecco’s modified Eagle’s medium (Nacalai Tesque) supplemented with 10% fetal bovine serum (FBS) purchased from BI (Kibbutz, Israel). HUVEC were purchased from Cell Applications (Cat No: 200 K-05 f, San Diego, CA, USA) and were maintained in endothelial cell growth medium from Cell Applications (San Diego, CA, USA). All cells were kept at 37 °C under 5% CO_2_ atmosphere.

### T7 phage display screen

A streptavidin-coated 96-well microplate (Nalge Nunc International, Wiesbaden, Germany) was washed with Tris-buffered saline (TBS) for 15 min followed by addition of 10 μM SQAP in TBS and of a vehicle control to each well and incubated for 1 h. Afterwards, the wells were washed three times with TBST (TBS, 0.1% Tween 20). An aliquot of the T7 phage library constructed from cDNA of lung cancer, HeLa or HUVEC cells was added to each well and the mixture was then incubated for 1 h. After incubation, the wells were washed 10 times with TBST. TBS containing 1% sodium dodecyl sulfate (SDS) was added as an elution buffer and incubated for 15 min to recover the remaining phage particles. In order to amplify the recovered phage particles, each eluate was mixed with a culture of *E. coli* BLT5615 purchased from Merck (Kenilworth, NJ, US) at an MOI of 0.001. The cells were then cultured at 37 °C until cell lysis was observed. The resulting solution was used for the next round of biopanning. The screening was repeated from five to six rounds for selection. In the final round, the eluate was mixed with 1 mL of BLT5615 culture and 10 mL of warmed top agarose and then poured across the surface of a lysogeny broth (LB)/carbenicillin plate. Once the top layer solidified, the plate was incubated at 37 °C for 3 h in order to allow the formation of phage plaques. A total of 16 plaques were randomly picked in each case and suspended in SM buffer (100 mM NaCl, 6 mM MgSO_4_, 20 mM Tris-HCl pH 8.0). The DNA sequence of each phage clone was then analyzed.

### Construction of expression vectors

The clones having 400–547 aa of SCP-2, 598–736 aa of MFE-2 and 910–1052 aa of FAK were used as templates for PCR. DNA encoding SCP-2 (400–547) was amplified using the following primer set: forward, 5′- CATATGGTAACACTCTACAAGATGGGTTTTCC-3′, reverse, 5′- CTCGAGTCAGAGCTTAGCGTTGCCTGGC-3′ (underlined bases point the restriction endonuclease recognition sites). For MFE-2 (598–736) DNA was amplified using the following primer set: forward, 5′- GAATTCATTTCAAATGCATATGTGGATCTTGCACCA-3′, reverse, 5′- CTCGAGTCAGAGCTTGGCGTAGTCTTTAAGAATCAT-3′. For FAK (910–1052) DNA was amplified using the following primer set: forward, 5′- CATATGCCCCCTCCTACTGCCAAC-3′, reverse, 5′- GGATCCTCAGTGTGGTCTCGTCTGCC-3′. The PCR product was inserted into pET-28a (+) expression (Merck, Kenilworth, NJ, US) vector in-frame with a His-tag at the N-terminus.

### Expression and purification of recombinants SCP-2 (400–547), MFE-2 (598–736) and FAK (910–1052)

The engineered SCP-2 (400–547), MFE-2 (598–736) and FAK (910–1052) expression vectors were used to transform *E. coli* Rosetta 2 (DE3) plysS (SCP-2 and MFE-2) or BL21 (DE3) (FAK) (Merck, Kenilworth, NJ, US). Bacterias were grown in LB medium containing 30 μg/mL of kanamycin and 100 μg/mL of chloramphenicol (SCP-2 and MFE-2) or 30 μg/mL of kanamycin (FAK) at 37 °C until the OD_600_ reached 0.5 before addition of 1 mM isopropyl thio-β-D-galactoside (IPTG). Then, the cells were incubated for 4 h at 37 °C. After incubation, the cells were harvested and suspended in binding buffer (7.8 mM Na_2_HPO_4_, 2.7 mM KCl, 1.5 mM KH_2_PO_4_, 0.5 M NaCl, 5% glycerol, 0.05% Triton X-100, pH 7.4) containing protease inhibitor cocktail (Nacalai Tesque, Kyoto, Japan). The cells were disrupted by sonication, and the resulting extract was clarified through centrifugation at 18,000 *g* 4 °C for 10 min. The clarified cell extracts were then filtered by a 0.22 μm filter (Millipore, Billerica, MA, USA). Recombinant SCP-2 (400–547), MFE-2 (598–736) and FAK (910–1052) were further purified by loading the samples onto a HisTrap HP column (1 mL, GE Healthcare, Little Chalfont, UK) using a fast protein liquid chromatographic system. Finally, bound proteins were eluted with a step gradient of imidazole at 0.3 mL/min flow rate using elution buffer II (binding buffer plus 0.5 M imidazole).

### SPR assay

The binding kinetics of SCP-2, MFE-2, ADRM1, HR23B and FAK with SQAP and SQP were analyzed on a Biacore 3000 instrument (GE Healthcare, Little Chalfont, UK). SCP-2 (400–547), MFE-2 (598–736) and FAK (910–1052) recombinant peptides were obtained as described in the previous section. The buffer of purified peptides was exchanged to HBS-P (150 mM NaCl, 0.005% Surfactant P20, 10 mM HEPES-NaOH pH 7.4) using a PD 10 column (GE Healthcare, Little Chalfont, UK) and the concentrations were quantified using DC protein assay kit (Bio-Rad Laboratories, Hercules, CA, USA). ADRM1 and HR23B human full length proteins were purchased. All proteins were immobilized onto the surface of a CM5 sensor chip using an amine coupling kit (GE Healthcare, Little Chalfont, UK). GST protein was immobilized on the control flow cell and the generated response units (RU) were subtracted from those of HR23B. Different concentrations of SQAP and SQP diluted in HBS-P (150 mM NaCl, 0.005% Surfactant P20, 10 mM HEPES-NaOH pH 7.4, 5% DMSO) were injected for 120 s at a flow rate of 20 μL/min at 25 °C. Kinetic parameters were determined by analyzing the data using BIAevaluation 4.1 software (GE Healthcare, Little Chalfont, UK).

### Docking simulation

The generation of docking models was performed using a fixed docking procedure in the Minimization and Dynamics protocols within DISCOVERY STUDIO (DS) 4.1 modeling software (Accerlys Inc.,San Diego, CA, USA). The calculations used a CHARMm force-field. The FAK (PDB entry: 1K05) was refined based on molecular dynamic simulations using standard dynamics cascade protocol in DS. The SQAP–FAK model was constructed with the Minimization and Dynamics protocols within DS.

### FAK phosphorylation level analysis

HUVEC cells were incubated for 20 h in serum-free medium in 60 mm dishes. SQAP, SQMG or SQP was added and incubated for 30 min followed by addition of 50 ng/mL VEGF (RD systems, Minneapolis, MN. USA) and incubation for another 30 min. FAK phosphorylation level was analyzed by Western blotting as described in the next section.

A549 cells were incubated in a serum-free medium for 48 h in 60 mm dishes (IWAKI, Tokyo, Japan). Then 10 μM SQAP, SQMG or SQP was added and incubated for 30 min followed by addition of fibronectin 10 μg/mL (Sigma, St. Louis, MI, USA) and incubation for another 30 min. FAK phosphorylation level was analyzed by Western blotting as described in the next section.

### Western blot analysis

Cells were harvested and solubilized in RIPA buffer (20 mM Tris-HCl, pH 7.5, 1% Sodium deoxycholate, 150 mM NaCl, 5 mM EDTA, 1% Triton X-100, 0.1% SDS, 0.1%, 1 mM PMSF, 1 mM vanadate and 1 μg/mL leupeptin). Protein quantification was determined using the DC protein assay kit (Bio-Rad, Hercules, CA, USA). A total of 15 μg of each protein sample were separated by SDS–PAGE gels and blotted onto polyvinylidene difluoride membranes (Merck, Kenilworth, NJ, US). After blocking for 1 h with Blocking-One (Nacalai Tesque, Kyoto, Japan), proteins were detected by incubating the membranes with the primary antibody at 4 °C overnight (anti-Y397 2:2000, anti-Y925 5:2000 and anti-FAK 0.5:2000) followed by incubation with horseradish peroxidase-conjugated secondary antibodies at room temperature for 1 h (anti-mouse 0.5:2000 and anti-rabbit 0.4:2000). The chemiluminescence was revealed performed using ECL Western blot detection reagents (GE Healthcare, Little Chalfont, UK) or ECL prime Western blot detection reagent (GE Healthcare, Little Chalfont, UK).

### Quantitative real-time RT-PCR analysis

Total RNA was prepared using an RNeasy Mini Kit (QIAGEN Inc., Hilden, Germany) according to the manufacturer’s instructions and then reverse-transcribed to cDNA with Transcriptor First Strand cDNA Synthesis Kit (Roche Applied Science, Mnnheim, Germany). Measurement of gene expression by quantitative analysis was performed using the LightCycler system (Roche Applied Science). Quantitative real-time RT-PCR analysis of *β*-actin, TSP-1 and VEGF-A expression were performed using the LightCycler FastStart DNA MasterPLUS SYBR Green I system (Roche Applied Science). The primer used for PCR were: *β*-actin, Forward: 5′-CACCAACTGGGACGACAT-3′, Reverse: 5′-ACAGCCTGGATAGCAACG-3′; TSP-1, Forward: 5′-GATGGAGAATGCTGAGTTG-3′, Reverse: 5′- TGAGGAGGACACTGGTAGAG-3′; VEGF-A, Forward: 5′-AGAGCAAGACAAGAAAATCC-3′, Reverse: 5′-TACAAACAAATGCTTTCTCC-3′. PCR amplification of the housekeeping gene, *β*-actin, was performed for each sample as control for sample loading and to allow normalization among samples. To determine the absolute copy number of the target transcripts, the fragments of *β*-actin or target gene amplified by PCR using the primer set were constructed with pGEM-T-easy cloning vector (Promega, Madison, WI). The concentrations of these purified plasmids were measured by absorbance at 260 nm and copy numbers were calculated from concentration of samples. A standard curve was created by plotting the threshold cycle (Ct) versus the known copy number for each plasmid template in the dilutions. The copy numbers for all unknown samples were determined according to the standard curve using LightCycler version 3.5.3 (Roche Applied Science). To correct for differences in both RNA quality and quantity between samples, each target gene was first normalized by dividing the copy number of the target by the copy number of *β*-actin, so that the mRNA copy number of the target was the copy number per the copy number of *β*-actin. The initial value was also corrected for the amount of *β*-actin indicated as 100% to evaluate the sequential alteration of the mRNA expression level.

### Wound healing assay

Wound healing assay was analyzed by microscope imaging. HUVEC or A549 cells were plated into 60 mm dishes and grown to confluence. The monolayer was wounded using the tip of a sterile 200-μl pipette (width: B1 mm). Debris was removed by washing twice with the serum-free medium. The cells were then allowed to migrate into the denuded areas for 11 h (HUVEC) or 25 h (A549) in a medium containing 1% FBS and VEGF (50 ng/mL) or fibronectin (10 μg/mL), respectively. Three images/well were acquired with a digital camera using a 5x objective attached to an Al Axio microscope (Zeiss, Oberkochen, Germany). Wound diameters in the images were measured and percentage of wound size was calculated as follows: [wound diameter at 11 or 25 h/wound diameter at 0 h] x 100.

Wound healing was additionally assessed in HUVEC or A549 cells using a cell migration kit (Oris, Madison, WI, USA) as described by the manufacturer. Cells were stained with CellTracker^TM^ Green (Life technologies, Carlsbad, CA, USA). The fluorescence emission signal in the migration zones was measured on a Wallac Arvo 5x microplate reader (Perkin Elmer, Waltham, MA, USA).

### Tumor xenograft

Cell lines and animal studies: A human lung small cell carcinoma, Lu-65, was obtained from the Cell resource Center for Biomedical Research (Sendai, Japan) and maintained in RPMI-1640 medium supplemented with 10% fetal bovine serum, 100 units/ml penicillin, 100 μg/ml streptomycin, and 1 mM sodium pyruvate at 37 °C in 5% CO humidified atmosphere. Viable tumor cell (1 × 106) were implanted subcutanceously into the right hind legs of male KSN nude mice (6–8 weeks old). After the tumor volume reached 50 to 100 mm3, the tumor were treated and the tumor growth was monitored by palpation. The size of the palpable tumors was measured using calipers every two days. The tumor volume (V, mm) was estimated based on the formula as follows; (minor axis)^2^ × (major axis) × 0.5.

Treatments: Synthesized SQAP was prepared one day before experimentation, and dissolved in saline at the appropriate concentrations. To analyze the tumor radioresponse, 2 mg/kg SQAP (dissolved in 100 μl of saline) was administered intravenously to the ‘SQAP’ group and ‘Combination’ group (adiministered five times daily from days zero to four). The same amount of saline was injected in the ‘control’ group and ‘X-irradiation’ group. Non-anesthetized mice were irradiated (8 Gy/fraction) on days zero and three with X-ray therapeutic machines HS-225 (225 kVp, 15 mA, 1.0 mm Cu filtration) (Shimadzu, Kyoto, Japan) while shielding the body with lead. All the experimental procedures were approved by the Experimental Animal Committee of Tokyo Medical and Dental University (No. 100015) and were performed in accordance with ARRIVE guidelines.

### Quantification of cell viability by WST-8 assay

HUVEC and A549 cells were seeded into a 96-well culture plate (2 × 10^4^ cells/well). The following day, cells were treated with SQAP or vehicle in a medium containing 1% FBS and VEGF (50 ng/mL) for 17 h (HUVEC) or fibronectin (10 μg/mL) for 46 h (A549). Cell viability was measured using Cell Count Reagent SF (Nacalai Tesque, Kyoto, Japan) following the manufacturer’s instructions. Briefly, 1/10 volume of the WST-8 solution was added to each well, and the plates were incubated at 37 °C for 1 h. Then, cell viability was determined colorimetrically by measuring OD450 using a microplate reader on a Wallac ARVO 5X microplate reader (Perkin Elmer, Waltham, MA, USA).

### Statistics

The data are reported as mean ± standard error of the mean (std) from at least three independent experiments. Statistical analysis was carried out using a student’s t-test.

## Additional Information

**How to cite this article**: Izaguirre-Carbonell, J. *et al.* Novel anticancer agent, SQAP, binds to focal adhesion kinase and modulates its activity. *Sci. Rep.* **5**, 15136; doi: 10.1038/srep15136 (2015).

## Supplementary Material

Supplementary Information

## Figures and Tables

**Table 1:** Phage display selection^a^.

Protein containing peptide	Frequency	Amino acid sequence	Location
Sterol carrier protein 2 (SCP-2)	9/16	VTLYKMGFPEAASSFRTHQIEAVPTSSASDGFKANLVFKEIEKKLEEEGEQFVKKIGGIFAFKVKDGPGGKEATWVVDVKNGKGSVLPNSDKKADCTITMADSDFLALMTGKMNPQSAFFQGKLKITGNMGLAMKLQNLQLQPGNAKL	400–547 aa (SCP-2 domain)
Peroxisomal multifunctional enzyme type 2 (MFE-2)	3/16	ISNAYVDLAPTSGTSAKTPSEGGKLQSTFVFEEIGRRLKDIGPEVVKKVNAVFEWHITKGGNIGAKWTIDLKSGSGKVYQGPAKGAADTTIILSDEDFMEVVLGKLDPQKAFFSGRLKARGNIMLSQKLQMILKDYAKL	598–736 aa (SCP-2 domain)
Proteasomal ubiquitin receptor (ADRM1)	7/16	PVPSAPAAASATSPSPAPSSGNGASTAASPTQPIQLSDLQSILATMNVPAGPAGGQQVDLASVLTPEIMAPILANADVQERLLPYLPSGESLPQTADEIQNTLTSPQFQQALGMFSAALASGQLGPLMCQFGLPAEAVEAANKGDVEAFAKAMQNKLAAALE	228–383 aa
UV excision repair protein (HR23B)	2/16	DPPQAASTGAPQSSAVAAAAATTTATTTTTSSGGHPLEFLRNQPQFQQMRQIIQQNPSLLPALLQQIGRENPQLLQQISQHQEHFIQMLNEPVQEAGGQGGGGGGGSGGKLAAALE	240–348 aa (ST1 domain)
Fokal adhesion kinase (FAK)	3/16	NSSPPPTANLDRSNDKVYENVTGLVKAVIEMSSKIQPAPPEEYVPMVKEVGLALRTLLATVDETIPLLPASTHREIEMAQKLLNSDLGELINKMKLAQQYVMTSLQQEYKKQMLTAAHALAVDAKNLLDVIDQARLKMLGQTRPH	910–1052 aa (FAT domain)

^a^After five to six rounds of selection by T7 phage display, 16 clones were randomly picked for DNA analysis to determine phage-displayed peptides.
