# Supplementary material for: Novel anticancer agent, SQAP, binds to focal adhesion kinase and modulates its activity
Source: Sci Rep. 2015 Oct 12;5:15136. doi: 10.1038/srep15136 (PMC4601023; doi:10.1038/srep15136)
Supplement: Supplementary Information [file srep15136-s1.pdf]

Novel anticancer agent, SQAP, binds to focal adhesion kinase and modulates its activity

Jesus Izaguirre-Carbonell [a], Hirofumi Kawakubo [a], Hiroshi Murata [a], Atsushi Tanabe [b], Toshifumi Takeuchi [a], Tomoe Kusayanagi [a], Senko Tsukuda [a], Takeshi Hirakawa [a], Kazuki Iwabata [a], Yoshihiro Kanai [a], Keisuke Ohta [c], Miura Masahiko [c], Kengo Sakaguchi [a], Sachihiro Matsunaga [a], Hiroeki Sahara [b], Shinji Kamisuki [a,*], Fumio Sugawara [a,*]

Supplementary Fig. S1 Tumor xenograft.

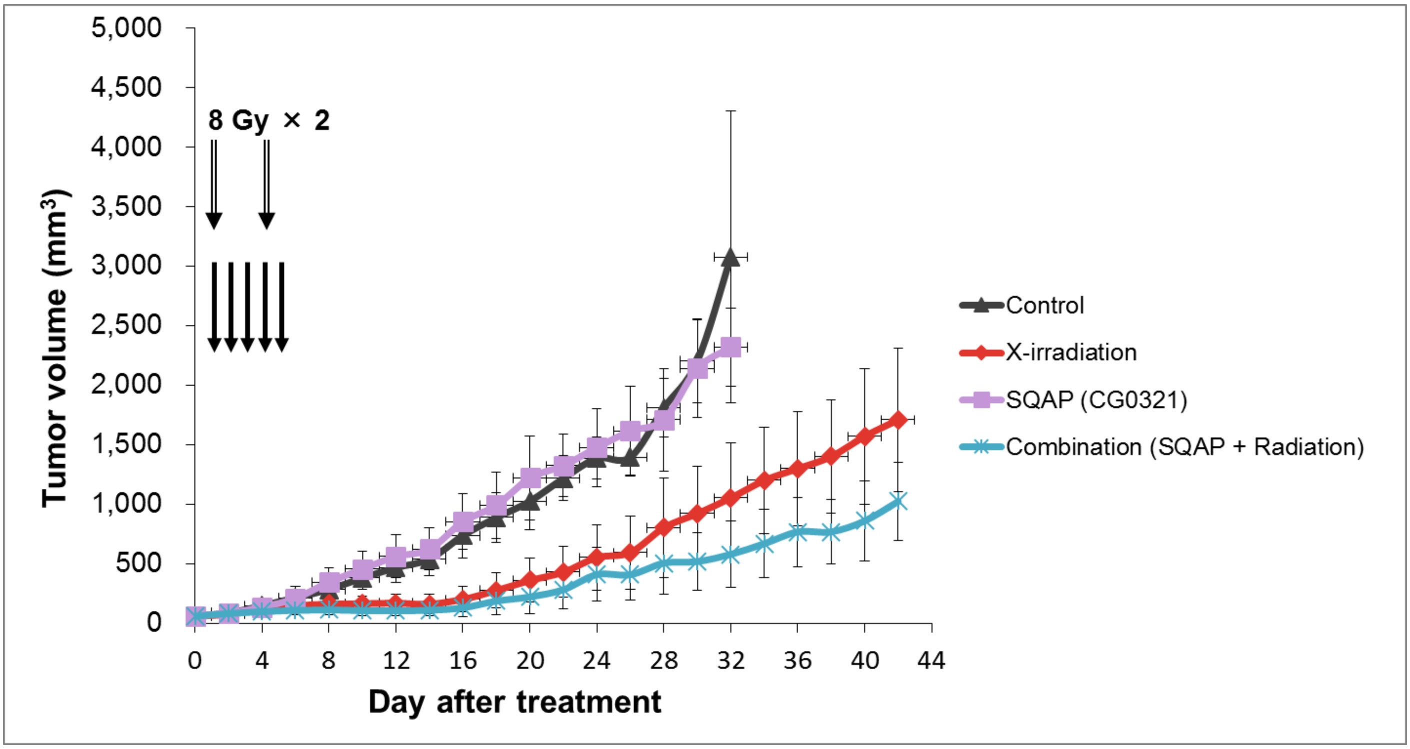

Relative tumor volume as a function of the days after initiating treatment. Five injections of SQAP (2 mg/kg, i.v.) plus two fractions of X-irradiation (8 Gy/fraction) were administered. Four kinds of treatments, Vehicle, SQAP (2 mg/kg/injection), X-irradiation (8 Gy/fraction), and Combination with SQAP and X-irradiation, were represented. Arrows indicate injections. Each point represents the mean±SD of nine tumors.

Supplementary S2 Fig. Phage display round selection.

A

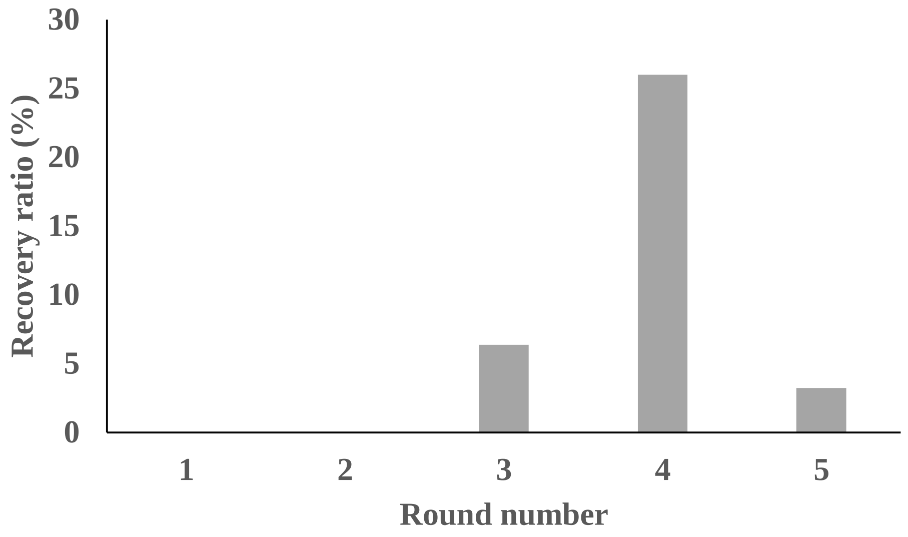

B

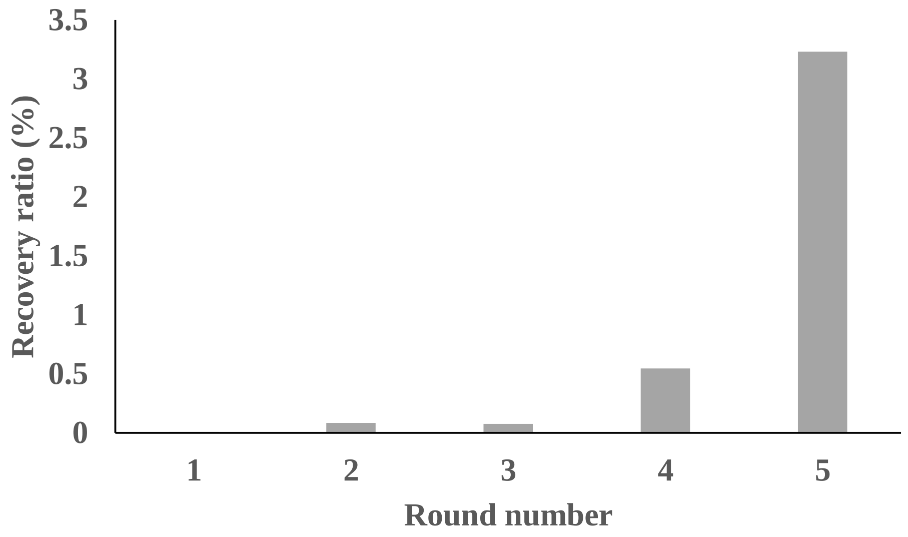

C

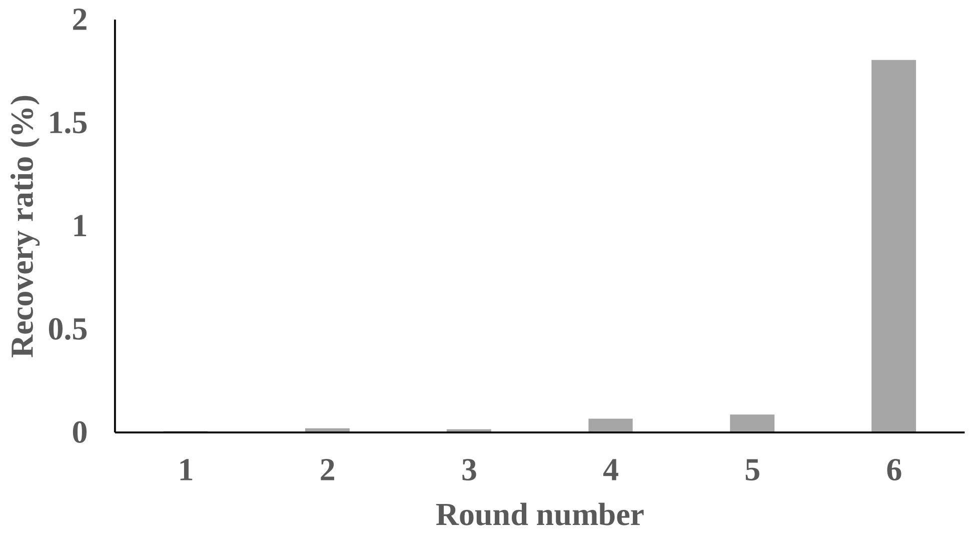

Relative enrichment of T7 phage particles binding to SQAP in each round of selection. The recovery ratio represents the phage titer in the eluate from the Bio-SQAP immobilized well compared with the input library titer. (**A**) Lung, (**B**) HeLa, (**C**) HUVEC phage libraries.

Supplementary S3 Fig. Effect of SQAP on Akt and paxillin phosphorylation

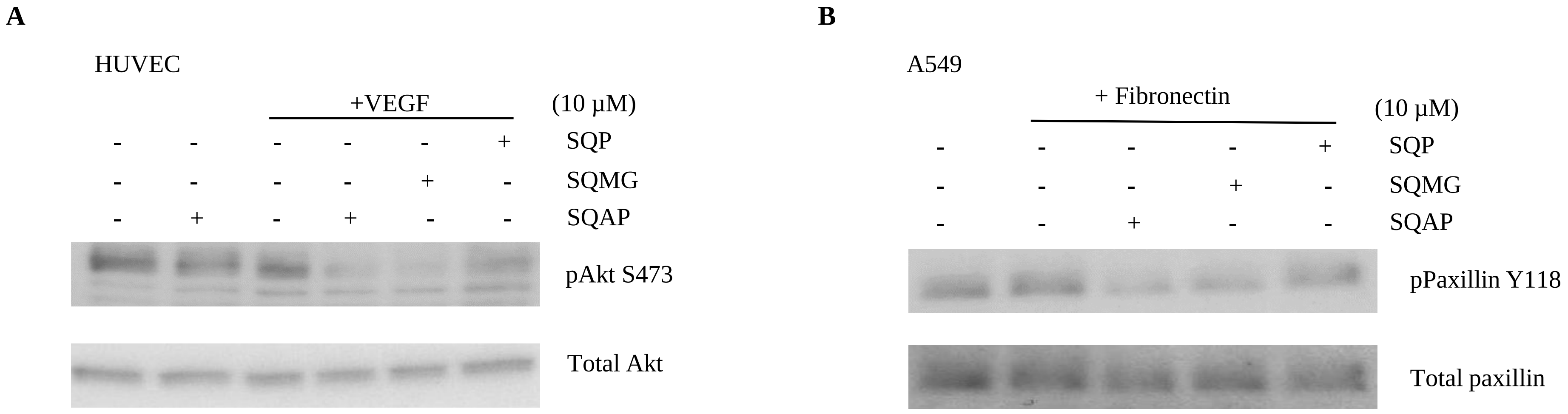

(**A**) SQAP was applied to HUVEC cells for 21 h before adding VEGF (50 ng/mL) for 30 min. (**B**) SQAP was applied to A549 cells for 21 h before adding fibronectin (10 µg/mL) for 30 min.

Supplementary S4 Fig. SQAP does not affect cell viability.

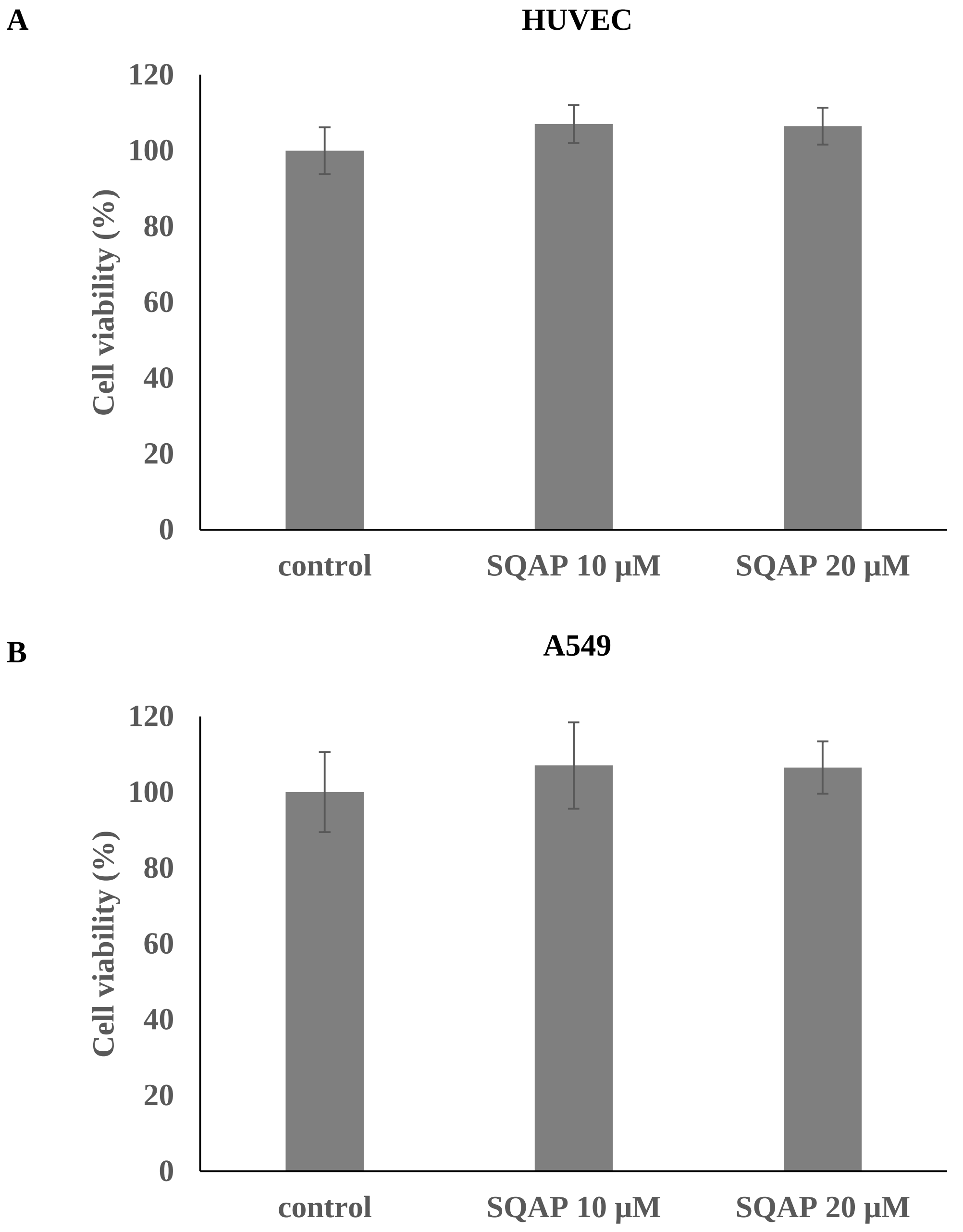

Live cells were measured with SF reagent under similar conditions as described in the wound healing assay in Fig. 5. (**A**) HUVEC and (**B**) A549 cells.
